# Belatacept in Kidney Transplantation: Reflecting on the Past, Shaping the Future

**DOI:** 10.3389/ti.2025.14412

**Published:** 2025-05-20

**Authors:** Johan Noble, Juliette Leon, Arnaud Del Bello, Dany Anglicheau, Gilles Blancho, Simon Ville, Lionel Couzi, Philippe Grimbert, Yannick Le Meur, Bruno Moulin, Nassim Kamar, Lionel Rostaing, Florence Herr, Antoine Durrbach, Dominique Bertrand

**Affiliations:** ^1^ Nephrology, Hemodialysis, Apheresis and Transplantation, Centre Hospitalier Universitaire (CHU) Grenoble-Alpes, La Tronche, France; ^2^ University Grenoble Alpes, Centre National de la Recherche Scientifique (CNRS), Institut National de la Santé et de la Recherche Médicale (INSERM), Centre Hospitalier Universitaire (CHU) Grenoble Alpes, Institute for Advanced Biosciences (IAB), Grenoble, France; ^3^ Service des Maladies du Rein et du Métabolisme, Transplantation et Immunologie Clinique, Hôpital Necker, Assistance Publique Hôpitaux de Paris (AP-HP), Paris, France; ^4^ Département de Néphrologie et Transplantation d’Organes, Centre Hospitalier Universitaire (CHU) Toulouse, Institut National de la Santé et de la Recherche Médicale (INSERM) UMR 1291, Toulouse Institute for Infectious and Inflammatory Diseases (INFINITY), Université Paul Sabatier, Toulouse, France; ^5^ Service de Néphrologie - Immunologie Clinique, Centre Hospitalier Universitaire (CHU) Nantes, Nantes Université, Nantes, France; ^6^ Institut de la Transplantation Urologie-Néphrologie (ITUN), Institut National de la Santé et de la Recherche Médicale (INSERM), Center for Research in Transplantation and Translational Immunology, UMR 1064, Nantes, France; ^7^ Department of Nephrology, Transplantation, Dialysis and Apheresis, Bordeaux University Hospital, Bordeaux, France; ^8^ Centre National de la Recherche Scientifique (CNRS)-UMR 5164 ImmunoConcEpT, Bordeaux University, Bordeaux, France; ^9^ Assistance Publique Hôpitaux de Paris (AP-HP), Service de Néphrologie et de Transplantation Rénale, Fédération Hospitalo-Universitaire, Innovative Therapy for Immune Disorders, Centre Hospitalier Universitaire (CHU) Henri Mondor, Créteil, France; ^10^ Immunorégulation et Biothérapie, University of Paris-Est-Créteil, Créteil, France; ^11^ Department of Nephrology, University Hospital La Cavale Blanche, Université de Bretagne Occidentale, Brest, France; ^12^ Service de Néphrologie-Dialyse-Transplantation, Hôpitaux Universitaires de Strasbourg, Strasbourg, France; ^13^ Service de Néphrologie, Transplantation, Hôpital Creteil, Assistance Publique Hôpitaux de Paris (AP-HP), France UMR1186, Institut Gustave Roussy, Université Paris-Saclay, Villejuif, France; ^14^ Department of Nephrology, Transplantation and Hemodialysis, Rouen University Hospital, Rouen, France

**Keywords:** belatacept, kidney transplantation, opportunistic infections, donor-specific antibodies, eGFR

## Abstract

Calcineurin inhibitors (CNIs) are a cornerstone of post-transplant immunosuppressive regimens. However, their use is associated with adverse effects, most notably chronic nephrotoxicity, which remains a leading cause of long-term allograft dysfunction. Belatacept, a selective costimulation blocker, offers a promising alternative to CNIs by aiming to reduce nephrotoxicity while maintaining efficacy in preventing acute rejection. While its use in *de novo* transplantation has been associated with improved graft and patient survival, it has also been linked to a higher incidence of acute rejection. Early post-transplantation conversion to belatacept has demonstrated significant improvements in renal function (eGFR gains ranging from +8.8 to +38.2 mL/min/1.73 m^2^ at 1 year post-conversion) but carries a higher risk of opportunistic infections. Late conversion protocols, typically initiated beyond 6 months post-transplantation, have shown sustained—although less pronounced—eGFR improvements and better long-term graft survival compared to CNI-based regimens. Additionally, belatacept appears to reduce the incidence of donor-specific antibodies. Future directions for the use of belatacept need further exploration, including its role in rescuing poor renal function, its combination with low-dose CNIs, mTOR inhibitors, or tocilizumab, and its application in desensitization protocols. By potentially striking a balance between efficacy and safety, belatacept may redefine the future landscape of transplant immunosuppression.

## Introduction

Calcineurin inhibitors (CNIs), particularly tacrolimus, are the most commonly used immunosuppressive agents to prevent rejection following solid-organ transplantation. Tacrolimus, in combination with mycophenolate mofetil (MMF) and steroids, forms the foundation of maintenance therapy for the majority of transplant recipients. This regimen has proven to be highly effective, with biopsy-proven acute rejection (BPAR) rates of approximately 8%–12% within the first year after kidney transplantation (KT) [1, 2]. However, tacrolimus is associated with several adverse effects, including an increased risk of diabetes, hypertension, and dyslipidemia. Moreover, tacrolimus contributes to both acute and chronic nephrotoxicity. Acute nephrotoxicity, which is reversible, results from hemodynamic changes due to afferent arteriolar vasoconstriction. In contrast, chronic nephrotoxicity is irreversible and leads to progressive decline in kidney function, characterized by interstitial fibrosis, tubular atrophy, chronic glomerulopathy, and vascular thickening.

**FIGURE 1:**
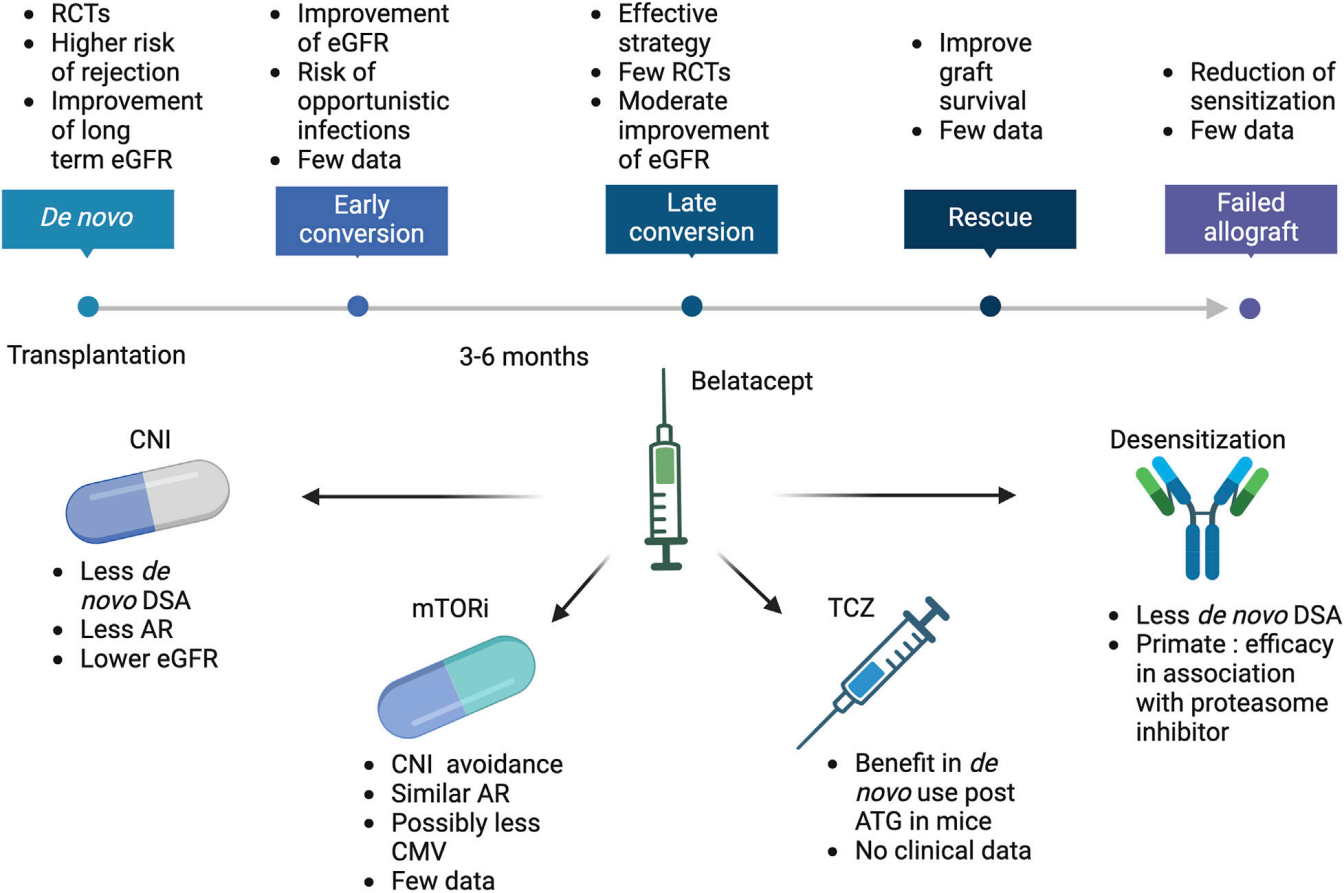
Belatacept current and future use in kidney transplantation. ATG, Antithymoglobulin; AR, Acute rejection; CMV, Cytomegalovirus; CNI, calcineurin inhibitors; DSA, Donor specific antibodies; eGFR, estimated glomerular filtration rate; mTORi, mammalian Target of Rapamycin inhibitors; RCTs, randomized controlled trials; TCZ, Tocilizumab.

The challenge to preserving long-term function is to find an immunosuppressive regimen that is as effective as tacrolimus in BPAR prevention but is not associated with chronic nephrotoxicity. Belatacept is the most advanced therapy in this field. Belatacept is a biotherapy derived from CTLA4-Ig (2 additional point mutations) with a higher avidity for CD80/CD86. It inhibits T-cell activation by impairing the CD28 pathway, the second signal for T-cell activation. CD28 is expressed by naive T cells and is involved in T-cell activation, proliferation, and survival in the presence of the TCR/CD3 signaling. Belatacept also interacts with CD80/CD86 on B-lymphocytes, impairing the maturation of naïve B cells in a transitional phenotype. Belatacept has been developed to replace CNI in *de novo* KT and to be used in combination with MMF and steroids. Phase II and III studies have demonstrated a significant improvement in renal function compared to cyclosporine A. In standard kidneys from brain-dead donors or living donors, the gain is up to 21 mL/min/1.73m2 at 3 years and is associated with an increased graft and patient survival [3]. In extended criteria donors, the gain is +11 mL/min/1.73 m^2^ at 3 years post-KT [4]. It also reduces the risk of *de novo* diabetes mellitus and improves cardiovascular risk factors [5]. The gain in renal function in *de novo* KT patients has led to exploring the use of belatacept as a replacement for CNI-treated patients to improve their renal function. The results of conversion strategies and the emerging use of belatacept are presented and discussed in this review (Figure 1).

## Benefit on Renal Function

### Early Conversion to Belatacept

The majority of studies have enrolled patients after 6 months post-transplantation. Few early conversions, i.e., before 6 months, have been reported. Initially, KT recipients were switched early to belatacept in the context of severe renal dysfunction. Two studies assessed the results in patients with very low eGFR (8 ± 12 mL/min/1.73 m^2^ (n = 25 patients) and 16 ± 12 mL/min/1.73 m^2^ (n = 20 patients) after a median time of 71 [15–161] and 42 [18–74] days post-KT respectively [6, 7]. The benefit in terms of eGFR at 1-year post conversion ranged between +16.6 mL/min/1.73 m [2] and +38.2 mL/min/1.73 m [2]. In the first study (Le Meur et al.), 48% of patients had a baseline eGFR <15 mL/min/1.73 m [2] and 29.1% were on dialysis. At 1 year, only 3 patients were still on dialysis. Graft and patient survival at 1 year were 83.3% and 96% respectively. In the second study (Wojciechowski et al.), 75% of patients required dialysis post-KT and before conversion. At 1 year post conversion, graft survival was 95% and no patient was still on dialysis. Patient survival was 100% at 1 year.

Some other non-randomized studies reported results on stable transplant patients in larger numbers of patients (60–453 patients) [8–12]. eGFR at the time of conversion in these patients ranged between 19.4 to 27 mL/min/1.73 m^2^. The gain of eGFR at 1 year post conversion ranged from +14.4 mL/min/1.73 m^2^ to +18.6 mL/min/1.73 m^2^. Graft and patient survival at 1 year were 83.3% and 97.2% (Bertrand et al.) and 100% and 90.9% (Moein et al.) respectively.

In a randomized controlled study, Tawhari et al. assessed the impact of early belatacept conversion (3 months) in 27 KT recipients with stable renal function (mean eGFR at conversion was 68.5 ± 18 mL/min/1.73 m [2]) [13]. Nine patients received belatacept with MMF, 8 received belatacept with low-dose tacrolimus, and 10 had no belatacept conversion. The evolution of eGFR at 2 years was +8.8 mL/min/1.73 m [2] in the belatacept plus low-dose tacrolimus patients, −0.38 mL/min/1.73 m^2^ in the tacrolimus plus MMF group and −6.60 mL/min/1.73 m^2^ in the belatacept plus MMF group. The rate of graft and patient survival was not different between groups (96.3% and 92.5% respectively at 2 years).

Overall, in the early post-KT period, conversion to belatacept appears to be associated with an important improvement in renal function, with acceptable graft and patient survival. The gain in GFR appears to be even higher in patients with delayed graft function in the very early phase. Further randomized studies are required to confirm the optimal use of belatacept during this period.

### Late Conversion to Belatacept

The renal function benefit of late conversion protocols has been demonstrated in several studies. In a randomized phase II trial of 173 patients comparing belatacept conversion to CNI at 19–20 months post-KT [14], the increase in eGFR at 36 months was +8.9 mL/min/1.73 m^2^ in the belatacept group compared to +1.1 mL/min/1.73 m^2^ in the CNI group (p = 0.01) [15, 16].

Budde et al. conducted a prospective randomized controlled study of belatacept conversion (n = 223) versus CNI maintenance (n = 223) at 6 months post-KT [17]. At 24 months, patient and graft survival (>97%)were similar in the 2 groups. At 24 months, the mean eGFR gain was +5.2 mL/min/1.73 m^2^ in the belatacept group and −1.9 mL/min/1.73 m^2^ in the CNI group (delta 7 mL/min/1.73 m^2^).

A recent retrospective study by Divard et al. compared 243 kidney recipients with a propensity-matched cohort of patients on a CNI-based regimen [18]. The median time to conversion was 1 year in the belatacept group, and the follow-up was 7 years. Graft survival was higher (78%) at last follow-up in the belatacept group versus 63% in the CNI group. The eGFR at 7 years was higher in the belatacept group, 26 mL/min/1.73 m^2^ versus 20.2 mL/min/1.73 m^2^ in the CNI group. Interestingly, a retrospective study evaluated the effect of conversion to belatacept in patients with severe vascular lesions (cv ≥ 2) and poor kidney function (eGFR between 25 and 27 mL/min/1.73 m^2^). The conversion to belatacept (n = 69) was found to be associated with a better graft survival at 3 years (84%) compared to patients who remained on CNI (n = 70, 65.1%) [19]. Fewer *de novo* DSA (7.4% versus 23.4%) but more opportunistic infections (OPIs) (7.6/100 person-years versus 1.0/100 person-years) were noted, while the rate of rejection and patient survival were similar.

Finally, the majority of patients switched to belatacept do not appear to have corticosteroids in their immunosuppressive treatments. A recent study compared 199 late-switched patients to belatacept without reintroduction of steroids versus 313 patients on concomitant steroids at the time of conversion [20]. The absence of steroids was not associated with an increased risk of PBAR or worse graft survival while the use of steroids was independently associated with worse patient survival.

## Risk of Rejection

Studies have shown that belatacept-based regimens are associated with an increased incidence of BPAR. The risk of acute rejection (AR) associated with the use of belatacept in KT varies depending on whether it is used in *de novo*, in early conversion, or in late conversion. Belatacept-resistant AR in KT involves different subsets of memory T cells, CD4^+^ CD28^+^ T effector-memory, CD8^+^ CD28null, and CD4^+^ CD57^+^ PD1-. These cells, particularly CD8^+^ T cells, exhibit high levels of IFN-γ production and granzyme B expression, indicating a robust cytotoxic response that is less susceptible to costimulatory blockade by belatacept but which can be regulated by mTOR inhibitors [21, 22]. Additionally, dysregulation of FOXP3+ regulatory T cells has also been implicated in belatacept-resistant AR [23].

Early conversion from CNI to belatacept also carries a risk of BPAR which varies from 5 to 22% at 2 years but was lower compared to *de novo* use [6, 7, 13]. Late conversion (after 6 months) to belatacept generally shows the lowest BPAR, varying from 4% to 8% [17, 18, 24].

Overall, the risk of AR is higher with *de novo* use and decreases with delayed conversion. The absence of antithymoglobulin use and the shorter delay between KT and belatacept conversion have been associated with an increased risk of BPAR [25]. To minimize the risk of AR during conversion, many authors have proposed an overlapping strategy with belatacept and a stepwise decrease of CNI within 1 or 2 months. The adopted scheme of CNI tapering varies, but generally involves a gradual reduction of CNI over a period of weeks to months, tailored to individual patient needs and clinical response. Nevertheless, this strategy is associated with a transient overimmunosuppression by inhibiting the first and second signals of T cell activation.

## Impact of Belatacept on Anti-HLA Antibodies and Antibody-Mediated Rejection

Despite a higher BPAR rate, the BENEFIT and BENEFIT-EXT trials showed a lower incidence of *de novo* DSA (1.4% and 3.8% in the more intensive belatacept group and 3.5% and 1.1% in the less intensive treatment group) and chronic rejection compared to a the cyclosporine groups (12.1% BENEFIT and 11.2% BENEFIT-Ext) [26]. Additionally, patients treated with belatacept had a significantly lower rate of IgM to IgG DSA conversion (22%) versus 65% in the cyclosporine group [27]. Compared to cyclosporine, the hazard ratio was 0.10, p < 0.001 for the more intensive belatacept group and 0.25, p < 0.001, for the less intensive group. These results correlate with the accumulation of transitional B cells in belatacept-treated patients suggesting an inhibition of their differentiation [28]. Samson et al. showed in a model of human germinal center formation in immunodeficient mice that belatacept inhibits the formation of these germinal centers [29]. They also showed a decrease in T follicular helper cells and B cells in the germinal centers in mice treated with belatacept, and a decrease in all types of immunoglobulin secretion. Belatacept is able to prevent the antibody response within the germinal centers [30].

Recently, 294 KT recipients on *de novo* belatacept (associated with 1 year of low-dose tacrolimus) were compared to 300 KT recipients who received long-term tacrolimus-based immunosuppression [31]. The rate of *de novo* class I and class II DSA at 1 year was not statistically different between the 2 groups (less than 4%). In subgroup analyses, based on the Eplet mismatch risk on DR/DQ, belatacept use was associated with a lower risk of immune events in intermediate-risk patients. At the last follow-up, the decrease in the DSA hazard ratio was 0.4 for the belatacept group.

For preexisting DSA in these cohorts, 100% and 94.5% of patients in the belatacept-treated groups had a decrease or stabilization of their DSA MFI compared to 71% in the cyclosporine groups [32].

Less data were available for conversion strategies. In the randomized conversion study by Budde et al., the prevalence of *de novo* DSA at 24 months was 1% in the group receiving belatacept and 7% in the CNI continuation group, whereas Kumar et al. did not find a significant decrease in DSA MFI post conversion in 19 patients switched at 44 months post KT [17].

## Opportunistic Infections and Tumors

The impact of belatacept on the risk of infection remains an essential area of investigation. In the BENEFIT and BENEFIT-EXT trials, infection rates, including serious infections and viral infections, did not significantly differ between groups [33, 34]. However, an increased risk of post-transplant lymphoproliferative disorder (PTLD) was observed, particularly in Epstein-Barr virus (EBV)-seronegative recipients. In randomized conversion studies, Budde et al. observed similar rates of infection between treatment groups, with one case of PTLD reported in the belatacept cohort [17]. Grinyó et al. reported no instances of PTLD in their phase 2 study [14]. These results support belatacept as a viable alternative for stable KT recipients on CNI therapy, provided careful monitoring and selection of EBV-seropositive patients.

Rescue conversion to belatacept in KT recipients is associated with a specific profile of OPIs. Several studies have documented the incidence of OPIs following belatacept conversion at a rate of 5.2–9.8 cases per 100 person-years [9, 10, 12, 35]. The most frequent OPIs were cytomegalovirus (CMV) infection and pneumocystis pneumonia, but other rare but severe infections include JC virus–induced progressive multifocal leukoencephalopathy and other viral or fungal infections. The comparative risk of OPIs is higher in belatacept-treated patients than in those maintained on CNI-based regimens, particularly for CMV reactivation and fungal infections [19]. Similarly, the incidence of pneumocystis pneumonia is higher in belatacept recipients without sufficient prophylaxis [9]. Several factors influence the risk of OPIs in patients switched to belatacept including baseline eGFR below 25 mL/min/1.73 m^2^ at the time of conversion, previously treated episodes of AR, duration of pre-existing CNI therapy and the overall immunological vulnerability of these patients [9]. OPIs contribute to substantial morbidity and mortality in this population, with infection-related deaths reported in up to 26.5% of cases and graft loss in 11.8%. Hospitalizations due to infections are also markedly higher in belatacept-treated patients, particularly in those who switch early [12]. Despite these risks, the overall graft and patient survival rates are acceptable, highlighting the need for robust infection prevention strategies.

Early conversion is associated with a substantially increased risk of CMV DNAemia and disease [12]. For instance, CMV DNAemia was reported in 31.6% of early converters compared to 11.5% of late converters [12]. In the *de novo* use of belatacept, Karadkhele et al. showed in high-risk CMV D+/R-recipients that belatacept-treated patients had a higher incidence of CMV viremia (50% of patients) compared to those treated with tacrolimus within 2 years of transplantation [36]. In the setting of rescue conversion, studies by Chavarot et al. and Bertrand et al. highlighted the heightened risk of CMV post-conversion [9, 37]. In both studies, valganciclovir was given 6 months post-transplantation to high-risk patients (D+/R-) and 3 months to intermediate risk patients (D+/R+ and D−/R+). Chavarot et al. reported that 17.9% of patients developed CMV disease after conversion, with a median onset of 9 months post-conversion [37]. The cumulative incidence of CMV disease was 6.6 per 100 person-years in belatacept-treated patients compared to 0.91 per 100 person-years in CNI-treated controls, representing a sevenfold increase. Bertrand et al. corroborated these findings by identifying CMV disease in 42.9% of OPIs in belatacept-treated patients [9]. CMV disease occurred primarily in high-risk (D+/R−) recipients, often after early conversion. Mortality associated with CMV disease was notable, accounting for 22.2% of deaths in patients with CMV disease.

Concomitant treatment could also play a role in the risk of OPIs following conversion. Chavarot et al. showed that steroids were independently associated with an increased risk of severe infections, including CMV disease [20]. ​The possible role of mTOR inhibitors in combination with belatacept has been highlighted as a strategy to mitigate CMV risks. In their recently published review, Zuber et al. emphasized the multifactorial nature of CMV risk, the importance of individualizing prophylaxis strategies, and the need for vigilance in high-risk patients [38].

Belatacept-treated KT recipients demonstrate a markedly reduced response to vaccination, including SARS-CoV-2 mRNA vaccines [39–41]. This reduced immunogenicity, both humoral and cellular, highlights critical challenges in protecting this vulnerable population during pandemics such as COVID-19 [42].

## Future Potential Use of Belatacept

### Belatacept in Combination With Tacrolimus

To address the increased rates of AR associated with standard belatacept regimens compared to CNI-treated patients, a combined strategy with short-term tacrolimus use in addition to belatacept has emerged in KT recipients. A cohort analysis of 50,244 patients including 417 patients receiving belatacept plus tacrolimus, 458 receiving belatacept, and 49,369 receiving tacrolimus has shown that the rate of AR was similar in tacrolimus and tacrolimus plus belatacept-based regimens and lower than in the belatacept regimen alone [43]. In contrast, eGFR and NODAT were higher and lower, respectively in the tacrolimus plus belatacept-treated patients than in the tacrolimus-treated patients. Results from a non-randomized study compared the modified belatacept-tacrolimus regimen (n = 87) with standard belatacept (n = 97) and tacrolimus treatments (n = 205) [44]. Patients also received Basiliximab induction, MMF, and corticosteroids. In the modified regimen, tacrolimus was administered for 3 months before tapering. At 3 months, the AR rates were similar for belatacept-tacrolimus (15%) and tacrolimus (17%), but nearly twice as high for belatacept (38%). However, the AR rate at 12 months for belatacept-tacrolimus (33%) was between that of tacrolimus (20.5%) and belatacept (50.5%). The rates of Banff grade IIB or III AR were 5%, 4%, and 13%, respectively. Despite higher AR rates, graft and patient survival at 3 years were similar between groups. To overcome the relapsed rate of AR, the tacrolimus exposure was extended to 9 months before being tapered within 2 months [44]. The 12-month AR rate for belatacept-extended tacrolimus was lower than in the historical tacrolimus cohort (16% vs. 20.5%), with 4% of patients experiencing Banff grade IIB or III AR. Over 3 years, the mean estimated GFR was higher for both belatacept-tacrolimus regimens than for standard tacrolimus treatment. Viremia rates for CMV and BK virus were similar between regimens suggesting that a belatacept-based regimen with transient tacrolimus use may yield AR rates comparable to those of standard CNI-based regimens without increasing infectious risks. Moreover, in a recent retrospective study analyzing the risk of de novo DSA based on the donor-recipient eplet mismatch showed that the risk was lower in the group of patients that received belatacept plus a transient exposure to tacrolimuns (n = 294) compared to the patients that received a tacrolimus-based regiment (n = 294) (hazard ratio [HR] = 0.4). The rate of antibody mediated rejection and acute rejection were also lower (HR = 0.2 and 0.45 respectively) [31].

### Belatacept in Combination With mTOR Inhibitors

Mammalian target of rapamycin (mTOR) is a protein kinase that has a central role in the regulation of cell metabolism, immune function, proliferation and migration. Sirolimus and everolimus are 2 mTOR inhibitors (mTORi) approved for the prevention of organ rejection in transplant recipients. The combination of belatacept with mTORi is an interesting association, allowing to remove CNI-related nephrotoxicity and adding the potential benefits of mTORi, such as antitumor and potential anti-CMV activity [45].

A randomized controlled study conducted by Ferguson et al. compared the evolution of belatacept *de novo* associated with MMF (33 patients), with sirolimus (26 patients) and with a standard group receiving tacrolimus with MMF (30 patients) [46]. At 1 year, the rate of BPAR was 4% in the mTORi group, and the mean eGFR was 61.8 mL/min/1.73 m^2^. The safety profile, along with patient and graft survival was similar between groups. The recovery, post-antithymoglobulin injection, of peripheral blood CD4^+^, CD8^+^, memory CD4+ and regulatory T cells was also similar between the different groups.

In 2014, Kirk et al. assessed the outcome of 20 KT recipients from non-HLA identical living donors who received alemtuzumab induction therapy followed by *de novo* belatacept and sirolimus [47]. Patients were randomized to receive or not receive unfractionated donor bone marrow. Three patients were switched to MMF because of sirolimus-related side effects. At 1 year, no clinical or histological rejection occurred and the mean eGFR was 89 ± 3.5 mL/min/1.73 m^2^. Safety was also excellent with no admissions for infection or malignancy. Interestingly, 10 patients reduced their immunosuppressive therapy and seven of these experienced no rejection on belatacept monotherapy. Safety was good: five patients had spontaneously resolving EBV viremia and 1 patient had a CMV viremia that resolved after increasing the prophylaxis dose.

From a cellular point of view, memory T cells may lose the expression of CD28, and thus escape the effect of belatacept and are implicated in the high rate of rejection in *de novo* studies reported above. After induction with a depleting agent, there is a marked increase in effector memory and terminally differentiated effector memory cells CD28-CD57^+^CD8^+^ T cells. *In vitro* and *in vivo* studies have shown that mTORi are able to suppress the expansion and the differentiation of these cells and thus reduce the risk of belatacept-resistant rejection [48, 49]. These cells have been shown to be more frequent in patients with belatacept-resistant rejection with increased expression of the mTOR pathway [22]. In CD4^+^CD57^+^ T cells, the mTOR pathway was not downregulated in belatacept-resistant cells as compared to belatacept-sensitive cells [49]. Taken together, these data suggest an interesting additional effect of mTORi in targeting belatacept-resistant CD8^+^ and CD4^+^ T cells.

The association of belatacept and mTORi may also be considered in post-KT conversion from the belatacept-MMF regimen to the belatacept-mTORi regimen. Very recently, Del Bello et al. reported their experience in 35 patients who were switched from MMF to mTORi in combination with belatacept [50]. They showed a lower incidence of CMV DNAemia in this group (incidence of 0.035/month of exposure) as compared to a propensity-matched cohort of belatacept–MMF treated patients (incidence of 0.072/month of exposure).

### Belatacept in Combination With Tocilizumab

The use of a depleting agent may be beneficial in combination with the use of belatacept *de novo* to prevent belatacept-resistant rejections, as it reduces the rate of rejection when associated with CNI [51]. However, in clinical practice, antithymoglobulin failed to prevent these rejections [52]. In a mouse model, Muckenhuber et al. showed that antithymoglobulins induce an important pro-inflammatory cytokine release, including IL-6, and that blocking IL-6 in addition to a *de novo* belatacept regimen prevents the occurrence of belatacept-resistant rejection and prolongs graft survival [53]. This combination promoted intragraft immune regulation and increased regulatory T cells within the graft.

Additionally, Herr et al. showed that the CD4^+^CD57+PD1-memory T cell population, associated with belatacept-resistant rejection, had more IRF7 transcript (associated with Interferon-α (IFN-α) and IL-6 regulation) [54]. Inhibition of IL-6, along with type I IFN-α, reduced the proliferation of these belatacept resistant cells.

### Use of Belatacept as a Desensitizing Molecule

In *de novo* studies, belatacept is associated with a lower rate of *de novo* DSA occurrence. Non-human studies have also shown the effect of belatacept in impairing the class switching of B cells. In a situation of high risk of immunization patients returning to dialysis, several teams continue immunosuppressive therapy for variable periods of time to prevent sensitization that impairs access to another transplantation, despite the associated increased risks of toxicity and infection [55]. To reduce sensitization, Badell et al. tested in a randomized study the efficacy of using belatacept in this setting in 60 patients, compared to immunosuppressive discontinuation in 7 patients. They found that belatacept reduced the incidence of *de novo* DSA and prolonged its onset, with a comparable safety profile [56].

For patients who are already sensitized, several strategies have been proposed to reduce or eliminate anti-HLA antibodies. The majority of strategies target B cells or long-lived plasma cells. Rituximab, which mainly targets B cells has failed to demonstrate significant efficacy. Proteasome inhibitors are effective in targeting antibody-producing cells but a rebound of antibodies is often seen [57]. The association of belatacept in this setting may be of interest because of its effect on germinal centers and since long-lived plasma cells re-express CD28 [58–60]. In non-human sensitized primate models, this strategy was effective in preventing DSA rebound as compared to standard immunosuppression with tacrolimus and MMF [61–64]. The “dual targeting” combination of belatacept and proteasome inhibitor on germinal centers was tested to desensitize 4 highly-sensitized heart transplant candidates and in antibody-mediated rejection post KT [65, 66]. This strategy was able to reduce anti-HLA antibodies and DSA. After discontinuation of proteasome inhibitors, belatacept was able to prevent antibody rebound in the majority of patients. Circulating cell analysis showed a reduction in naïve and memory B cells and of T follicular helper cells.

## Conclusion

In summary, belatacept is emerging as a valuable therapeutic option in KT, demonstrating advantages such as improved renal function and a favorable long-term safety profile compared to CNI-based regimens. However, its association with an increased risk of acute rejection, particularly in *de novo* protocols or early conversion, highlights the need for individualized patient selection and close monitoring. Future studies are essential to refine the optimal use of belatacept to ensure the best balance between efficacy and safety in different transplant populations.

## Abbreviations

AR, Acute Rejection; BPAR, Biopsy Proven Acute Rejection; CKD, Chronic Kidney Disease; CNI, CalciNeurin Inhibitor; DSA, Donor-Specific Antibody; eGFR, estimated Glomerular Filtration Rate; HLA, Human Leukocyte Antigen; IFN-α, Interferon-α; KT, Kidney Transplantation; MMF, Mycophenolate Mofetil; mTORi, mammalian Target Of Rapamycin inhibitors.
